# The Use of Mock Standardized/Simulated Patient Encounters in Facilitating Development of Clinical Competence in Medical Students

**DOI:** 10.7759/cureus.40562

**Published:** 2023-06-17

**Authors:** Nga N Tran, Denisia Thomas, Chloe F Haverkamp, Emilee L Leslie, David Kashmer

**Affiliations:** 1 Simulation Center, Edward Via College of Osteopathic Medicine, Auburn, USA; 2 Chair of Surgery & Associate Dean for Simulation, Edward Via College of Osteopathic Medicine, Auburn, USA

**Keywords:** simulation in medical education, sp, mock, medical student, standardized patients

## Abstract

Background

Standardized patient (SP) encounters are used in medical school to mimic clinical practice by exposing students to possible clinical situations they may encounter in future practice. SP includes trained individuals who portray the roles of patients to help medical students practice recording medical histories, physical exam skills, communication skills, and typing a subjective, objective, assessment and plan (SOAP) note. The goal of SP is to prepare medical students adequately during their didactic years before beginning clerkships. SP encounters have become standard in medical school curriculums, but the option for a mock SP encounter has not.

Methods

In this study, a total of 34 participants completed an eight-question survey before a mock SP experience and after their graded SP encounters to assess the students' confidence levels and preparedness. Each question addressed a different aspect of the student’s satisfaction and attitude regarding the SP encounters. The answers were measured on a Likert scale from 1 (not prepared) to 5 (very prepared). The central tendency (mean) was analyzed using a T-test with Welch's method. The standard deviation was analyzed using Bonett's test. A Cronbach’s alpha was used to show the reliability of the survey used.

Results

The first four questions addressed the student’s satisfaction with their mock SP experience. The mean student satisfaction with being able to ask questions to improve their knowledge and understanding improved, with a p-value of < 0.001. Student satisfaction with having the opportunity to record a patient’s history improved, with a p-value of < 0.001. The overall satisfaction with having a chance to practice physical exam skills improved, with a p-value of < 0.001. Mean student satisfaction with practicing treatment and counseling of patients improved, with a p-value of <0.001. The final four questions addressed the students' attitudes regarding their experience. The mean confidence of students improved, with a p-value of <0.001. Students also reported an improved attitude towards the feedback they received, with a p-value of <0.001. The final two questions did not show a statistically significant difference in answers. Students rated the final two questions equally before and after the mock SP experience, with p-values > 0.05. These questions addressed whether mock SP encounters would be beneficial and asked if they wanted additional mock simulation opportunities built into the curriculum.

Conclusions

The students showed improved confidence, attitudes, and satisfaction surrounding standardized patient encounters. The results supported the hypothesis that there would be a difference between the responses before and after the SP encounter. The questionnaire showed that the students reported subjective changes in their competence following the encounter.

## Introduction

Medicine and healthcare are constantly changing, and numerous recent advances aim to make the physician’s job easier. These advances include improvements in technology and media to allow for easy accessibility of information and medical charts [1]. However, this can cause a hindrance to physician-patient relationships since physicians will focus more on the computer in the room than on the patient. Therefore, there is an emphasis on adopting a more patient-centered approach [2]. This creates a motion to create new and inventive ways to help medical students prepare for future patients. Standardized patients (SP) have been incorporated early in the medical school curriculum to instill the concept of a patient-centered approach. SP is a simulation-based learning approach that exposes medical students to possible clinical situations that they may experience in future practice. SP includes trained individuals who portray the roles of patients to help medical students practice history-taking, physical exam skills, communication skills, and subjective, objective, assessment and plan (SOAP) note writing. Medical students use SP encounters as graded training sessions for real-patient encounters where students can learn from their mistakes in a safe learning environment [3].

Although SP encounters are emphasized in the curriculum, medical students don’t always feel prepared due to time constraints and a lack of practice [4]. This causes medical students to lose confidence in their clinical skills. A mock SP can serve as an ungraded practice run to help medical students prepare for their graded SP. This SP practice experience can give students the opportunity to develop their clinical judgment for patient assessment and treatment, thus allowing them the repetition necessary to be effective in the patient encounter scenario. Medical students who participate in a mock SP may feel less anxious and more prepared for their graded SP experience.

## Materials and methods

This research is a quality improvement project, and its purpose is to assess whether participating in a mock SP aids medical students in feeling more prepared for their required standardized patient encounters. A mock SP was provided by the Simulation Center at the Edward Via College of Osteopathic Medicine in Auburn, Alabama (VCOM), allowing students to practice and hone their skills while getting feedback without the pressure of being graded. First-year medical students at VCOM participated in this study. Simulation (SIM) interns from the simulation center were trained to serve as the patients in a practice case. A SIM intern is a second-year medical student who operates as a tutor for clinical skills and helps students improve their interview skills prior to the graded SP experience.

An initial email was sent to first-year medical students at VCOM to introduce the optional mock standardized patient encounter and to provide a date and time for when the sign-up link would be available. A second email was sent a week later with the sign-up link. Using a random number generator, 34 subjects were obtained. Each student completed a pre-mock SP survey on the day of the mock SP encounter. Each mock SP encounter consisted of 30 minutes with a SIM intern: 20 minutes to obtain a history and perform a physical exam, and 10 minutes for feedback. 12 SIM interns participated, and each SIM intern completed mock SP encounters with a maximum of four students throughout the day. At the conclusion of the graded SP encounter, the students who participated in the mock SP encounter filled out a post-graded encounter survey to determine the effectiveness of the mock encounter. The pre- and post-surveys included the same eight questions. The first four questions addressed student satisfaction and were as follows: I was able to ask questions to improve my knowledge and understanding; I had the chance to work on patient history taking; I had the chance to work on my physical exam skills; I had the chance to work on treatment or counseling. The final four questions addressed student attitude and were as follows: I felt more confident during a standardized patient encounter; I felt like the feedback provided was constructive; I thought a mock simulation would be a good way of learning clinical skills; I wanted more mock simulation opportunities in the curriculum. The survey tools distributed to participating students can be found in the appendices.

Each question addressed a different aspect of the student’s satisfaction and attitude regarding the SP encounters to determine whether an ungraded mock SP will benefit medical students' clinical competency. The Likert scale utilized continuous data and ran from 0 to 5. The students were able to rate how much the mock SP prepared them, from 0 (not prepared) to 5 (very prepared). A visual analog scale was used, which allowed students to pick a more accurate scale ranging from 0 to 5. The central tendency (mean) was analyzed using a T-test with Welch's method. The standard deviation was analyzed using Bonett’s test.

## Results

A total of 34 first-year medical students at VCOM participated in this study. The central tendency (mean) was analyzed using a paired T-test. The students reported being able to ask questions to improve their knowledge and understanding with a pre-intervention mean of 2.64 (CI: (2.27, 3.02)), a post-intervention mean of 4.58 (CI: (0.43, 0.75)), a mean difference of -1.94, and a p-value < 0.001, which can be concluded that there is statistical significance in means. The students rated their chance to work on patient history with a pre-intervention of 2.63 (CI: (2.26, 2.99)), a post-intervention mean of 4.69 (CI: (4.51, 4.88)), a mean difference of 2.07, and a p-value <0.001, so it can be concluded that the difference between means is significant. The participants rated the chance to work on physical exam skills with a pre-intervention mean of 2.55 (CI: (2.17, 2.92)), a post-intervention mean of 4.37 (CI: (4.10, 4.73)), a mean difference of -1.82, and a p-value <0.001. The difference between means can be concluded to be significant. The students rated their chance to work on treatment or counseling patients with a pre-intervention mean of 2.02 (CI: (0.92, 1.68)), a post-intervention mean of 4.41 (CI: (4.07, 4.76)), a mean difference of -2.39, and a p-value <0.001, so the difference between means can be concluded to be significant. The students rated their confidence during a standardized patient encounter with a pre-intervention mean of 2.00 (CI: (1.60, 2.40)) and a post-intervention mean of 4.65 (CI: (4.44, 4.86)), a mean difference of -2.65, and a p-value <0.001, so the difference between the means can be concluded to be significant. The students were asked to rate if they felt like the feedback that they were provided was constructive. With a pre-intervention mean of 1.54 (CI: (1.28, 1.81)), a post-intervention mean of 4.72 (CI: (4.51, 4.92)), a mean difference of -3.17, and a p-value <0.001, one could conclude that there is a significant difference between the means. The students were evaluated on whether a mock simulation would be a good way of learning clinical skills. With a pre-intervention mean of 4.75 (CI: (4.57, 4.93)), a post-intervention mean of 4.82 (CI: (4.70, 4.94)), a mean difference of -0.07, and a p-value >0.05, there is not enough evidence to conclude that the means differ. The students rated whether they wanted more mock simulation opportunities in the curriculum. With a pre-intervention average of 4.60 (CI: (4.34, 4.86)), a post-intervention average of 4.87 (CI: (4.77, 4.98)), a mean difference of -0.27, and a p-value >0.05, there is not enough evidence to conclude that the two means differ at the 0.05 level of significance. These results are summarized below (Table 1).

**Table 1:** Results of the questionnaire * denotes p-values of statistical significance

	Mean before intervention	Mean post-intervention	Difference between means	P-value
Question 1	2.6406	4.5846	-1.944	<0.001*
Question 2	2.625	4.6949	-2.0699	<0.001*
Question 3	2.5461	4.3707	-1.8246	<0.001*
Question 4	2.0239	4.4136	-2.3879	<0.001*
Question 5	2.0018	4.6471	-2.6452	<0.001*
Question 6	1.5423	4.7151	-3.1728	<0.001*
Question 7	4.75	4.8235	-0.073529	>0.05
Question 8	4.342	4.8713	-0.26838	>0.05

The standard deviation (SD) was analyzed using Bonett's test. Question 1 had a pre-intervention SD of 1.08 (CI: (0.84, 1.47)), a post-intervention SD of 0.55 (CI: (0.43, 0.75)), and a p-value <0.05, so it can be concluded that the SD differ. Question 2 had a pre-intervention SD of 1.03 (CI: (0.82, 1.39)), a post-intervention SD of 0.54 (CI: (0.34, 0.92)), and a p-value <0.05, so it can be concluded that the SD differ. Question 3 had a pre-intervention SD of 1.07 (CI: (0.85, 1.43)) and a post-intervention SD of 0.53 (CI: (0.45, 0.80)) with a p-value <0.05, so it can be concluded that the SD differ. Question 4 had a pre-intervention SD of 1.05 (CI: (0.93, 1.59)), a post-intervention SD of 0.57 (CI: (0.47, 0.90)), and a p-value <0.05, so it can be concluded that the SD differ. Question 5 had a pre-intervention SD of 1.14 (CI: (0.97, 1.43)) and a post-intervention SD of 0.61 (CI: (0.33, 1.19)), p-value <0.05, so it can be concluded that the SD differs. Question 6 had a pre-intervention SD of 0.75 (CI: (0.48, 1.23)) and a post-intervention SD of 0.30 (CI: (0.16, 0.58)), p-value <0.05, so it can be concluded that the SD differs. Question 7 had a pre-intervention SD of 0.53 (CI: (0.28, 1.03)), a post-intervention SD of 0.34 (CI: (0.20, 0.63)), and a p-value >0.05, so it can be concluded that the SD do not differ. Question 8 had a pre-intervention SD of 0.76 (CI: (0.57, 1.09)), a post-intervention SD of 0.60 (CI: (0.29, 1.28)), and a p-value >0.05, so it can be concluded that the SD do not differ. These results are summarized below (Table 2).

**Table 2:** SD results of the questionnaire * denotes p-value of statistical significance SD: standard deviation

	SD before intervention	SD post-intervention	P-value
Question 1	1.08	0.55	<0.05*
Question 2	1.03	0.54	<0.05*
Question 3	1.07	0.53	<0.05*
Question 4	1.05	0.57	<0.05*
Question 5	1.14	0.61	<0.05*
Question 6	0.75	0.30	<0.05*
Question 7	0.53	0.34	>0.05
Question 8	0.76	0.60	>0.05

A Cronbach’s alpha was used to show the reliability of the survey. The alpha for the pre-mock SP survey was 0.74, which is considered to have "acceptable" reliability, and the alpha for the post-graded SP survey was 0.85, which is considered to have "good" reliability. The alpha coefficient of reliability ranges from 0 to 1. The higher the alpha, the more the items have shared covariance and measure the same underlying concept.

## Discussion

Clinical competence is a skill that may not come naturally to everyone, but it is a skill that medical students are evaluated on. The primary purpose of this study was to examine the effectiveness of mock standardized patient encounters in preparation for the actual graded encounter. The concept of standardized patients originated in the 1960s when a neurologist wanted to provide sufficient real-life teaching experience for his students [5]. It became a standard, common training tool in medical schools across the United States [6]. In a standardized patient encounter, a patient is simulated by an actor. The actor is assigned a case that could be based on an actual patient or a fabricated case. The students then determine the patient’s diagnosis and formulate possible treatment plans by obtaining a detailed history and physical exam (H&P) and associated labs and imaging provided by the instructors [7,8]. Students are graded and provided with feedback in most cases to allow for improvements.

Many medical schools across the United States have incorporated mock SP into the curriculum under the term OSCE, which stands for Objective Structured Clinical Examination [9]. They hold the same concept of allowing students to practice and demonstrate clinical skills in a simulated medical scenario and have become one of the most widely used methods of assessing aspects of clinical competency in healthcare education [10]. SPs and OSCEs both take place during the didactic years of medical school in preparation for clerkship rotations, which occur during the second half of medical school [11,12]. A 2020-2021 survey given by the AAMC (Association of American Medical Colleges) assessed the methods used for clinical knowledge and/or skills assessment in clinical experience. Out of the 155 medical schools that participated in the study, 152 reported that they utilized direct observation of students performing H&Ps by faculty or residents. Of the participating medical schools, 42 do not require a course for clerkships [13]. During the COVID-19 pandemic, the education of medical students became hindered due to limited social interactions. Medical schools began utilizing virtual standardized patient encounters to mimic telehealth experiences in clinical practice to provide a different educational modality [14]. This allowed students to still have methods of practicing clinical skills while maintaining social distancing. Earlier research shows the effectiveness of standardized patient encounters to prepare healthcare professionals for clinical experiences [15].

There was evidence of statistical significance in responses to all but two questions from the survey given before and after the SP encounter. The statistically significant change in those responses showed an elevated confidence and improved satisfaction that was not due to chance and was directly related to the opportunity to practice their skills in a non-graded environment. The two questions that did not have a statistically significant change in response asked the students if they believed a mock SP would be a good way to learn clinical skills and if they would want similar experiences in the future. The fact that there was not a statistically significant change in these answers shows that students expected the experience to be beneficial, and they were happy with their experience after completing the mock SP. In the findings stated in this report, the students expressed the importance of hands-on training throughout the medical school experience. From these results, it can be concluded that mock standardized SP encounters prior to a graded encounter contribute to the development of clinical competence in healthcare professionals.

Limitations of the study include bias due to the subjectivity of the answer and variations in the delivery of the mock SP among different SIM interns. Students may have had different knowledge levels regarding clinical skills based on past experiences. Many students enter medical school with a history of clinical experience either as a medical scribe, prior experience as a nurse or EMT, or volunteer experience.

## Conclusions

The present study shows that participation in mock standardized patient encounters that precede a graded standardized patient encounter contributes to the development and competence of healthcare professionals. When students evaluated their confidence before and after the mock, there was a clinically significant difference in the average, not simply due to chance. Although standardized patient encounters have become a customary practice in medical schools across the United States, mock encounters have not.

## Figures and Tables

**Figure 1:**
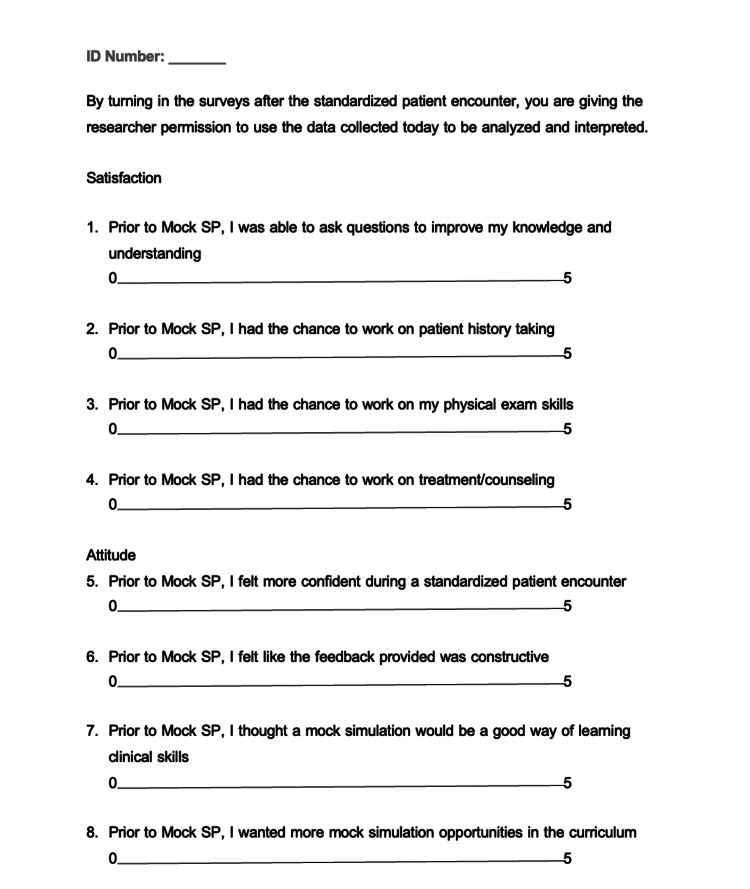
Pre-mock standardized patient survey SP: standardized patient

**Figure 2:**
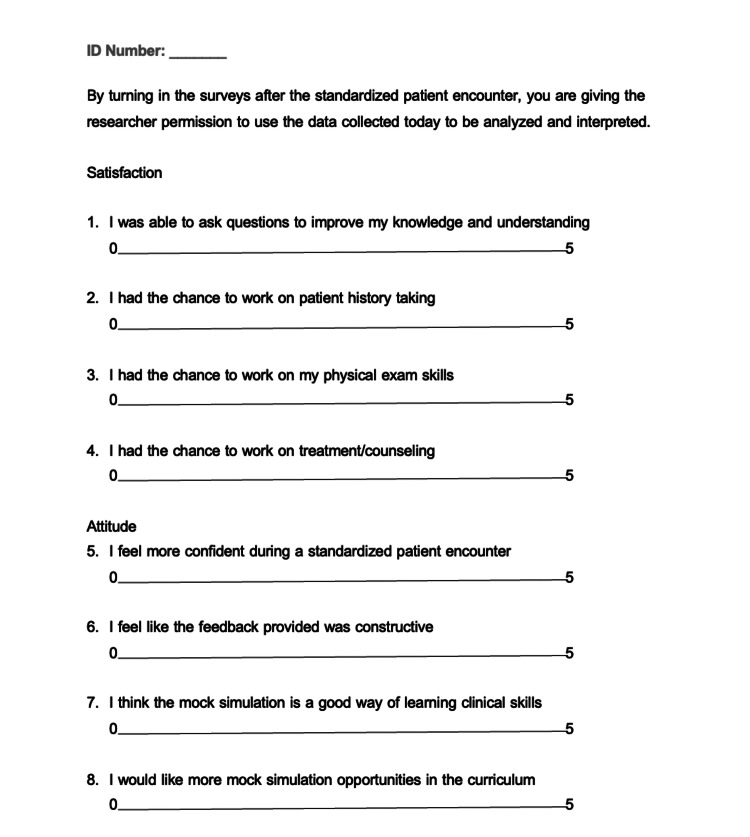
Post-graded standardized patient exam survey
